# A shallow water numerical method for assessing impacts of hydrodynamics and nutrient transport processes on water quality values of Lake Victoria

**DOI:** 10.1016/j.heliyon.2024.e25125

**Published:** 2024-02-03

**Authors:** Seema Paul, Benedict T.I. Reinardy, David D. Walakira, Prosun Bhattacharya, Henrik Ernstson, Zahra Kalantari

**Affiliations:** a Division of Water and Environmental Engineering, Department of Sustainable Development, Environmental Science and Engineering, KTH Royal Institute of Technology, Teknikringen 10B, 114 28, Stockholm, Sweden; b Numerical Analysis, CSC Skolan, KTH Royal Institute of Technology, Stockholm, Sweden; c Division of Strategic Sustainability Studies, Department of Sustainable Development, Environmental Science and Engineering, SEED, KTH Royal Institute of Technology, Stockholm, Sweden; d Mathematics Department, Makerere University, 7062, Kampala, Uganda

**Keywords:** Water system diagram, Vertically integrated SWE model, Raindrop diffusion, Flow evaluation, Wind hydrodynamics, Deposition assessment

## Abstract

Lake Victoria is the world's largest tropical lake and the third-largest water body, providing significant water resources for surrounding environments including the cultural, societal, and livelihood needs of people in its basin and along the White Nile. The aim of this study was to use decade-long time series of measured lake flow in the lake system and phosphorus deposition to develop a suitable numerical model based on shallow water equations (SWE) for assessing water quality in Lake Victoria, an increasingly important tool under climate variation. Different techniques were combined to identify a numerical model that included: i) a high-resolution SWE model to establish raindrop diffusion to trace pollutants; ii) a two-dimensional (2D) vertically integrated SWE model to establish lake surface flow and vertically transported wind speed flow acting on lake surface water by wind stress; and iii) a site-specific phosphorus deposition sub-model to calculate atmospheric deposition in the lake. A smooth (non-oscillatory) solution was obtained by applying a high-resolution scheme for a raindrop diffusion model. Analysis with the vertically integrated SWE model generated depth averages for flow velocity and associated changes in water level profile in the lake system and showed unidirectional whole lake wind blowing from the southwest to northeast. The atmospheric phosphorous deposition model enabled water value assessment for mass balances with different magnitudes of both inflows and outflows demonstrating annual total phosphorus at 13,500 tons concentrating at mid-lake western and eastern parts. The model developed here is simple and suitable for use in assessing flow changes and lake level changes and can serve as a tool in studies of lake bathymetry and nutrient and pollution transport processes. Our study opens towards refining models of complex shallow-water systems.

## 1 Introduction

Lake Victoria, also known by its indigenous name *Nalubaale*, the abode of the spirits (Hoesing, 2012), lies in a low-lying area between the raised rift shoulders of the eastern and western branches of the East African Rift System [1,2]. In comparison to the other Great Lakes of Africa (e.g., Tanganyika, Turkana, Nyasa), Lake Victoria is relatively young, formed through tectonic forces some 750,000 years ago [3] and went through periods of complete desiccation as recently as 12,000 years ago. The lake constitutes the primary freshwater input to the White Nile and contains much of the freshwater storage of East Africa. It is crucial for regional fisheries across East Africa and for preserving endemic native freshwater species [2,4]. Lake Victoria is the largest tropical lake in the world, but hydrologically it is also considered shallow relative to its large area, with an average depth of 40m and a maximum depth of 80m [5,6]. The total surface catchment is around $59,947\;{km}^{2\;}$ and the lake area $68,800\;{km}^{2}$ [7]. Lake Victoria lies within three East African countries: Kenya (6% of lake surface), Uganda (43% of lake surface) and Tanzania (51% of lake surface) [[8], [9], [10], [11]].

The aim of this study was to develop a hydrodynamic model for assessing water quality in Lake Victoria which can serve natural resource management and wider governance processes under increased climate variability. Such models can help to take into account the regional environmental context with significant variation of wind strength and temperature throughout the day. Average daily temperatures remain relatively constant throughout the year, with seasonal variations dominated by precipitation—with average monthly precipitation at more than 60mm [[8], [9], [10]]. The tropical climate of Lake Victoria has a significant influence on both the water levels and wind speed, which correspond to a hydrodynamic driving force for the water body. Thus, climate variability can have significant impacts on the hydrodynamics of the lake [12,13]. For sustainable future development of environmental water resources in the region, the ability to model the hydrodynamics of Lake Victoria will become increasingly important while also challenging due to the complex geo-meteorological processes that frequently occur in and around Lake Victoria with direct influence on hydrodynamics and the lake's associated ecosystems.

**Fig. 1:**
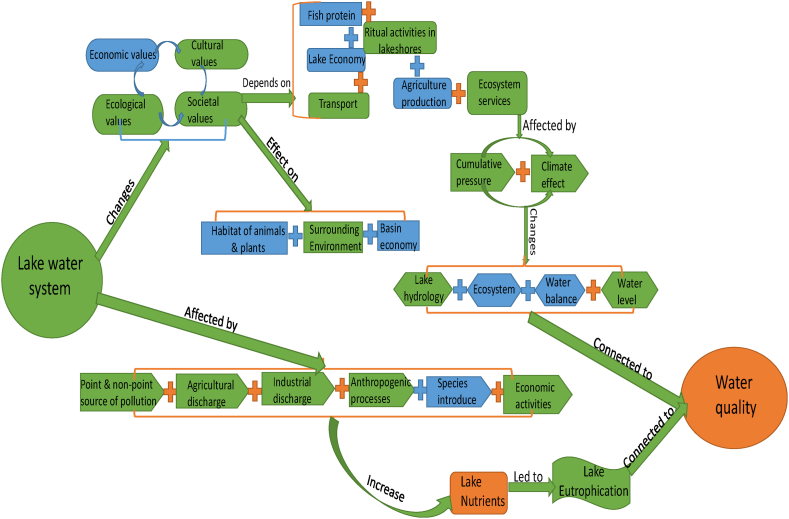
Environmental and social water values of Lake Victoria and influencing factors identified in previous studies (blue icons and arrows) and factors examined in the present study (orange and green icons). (For interpretation of the references to colour in this figure legend, the reader is referred to the Web version of this article.)

Lake Victoria is furthermore central to millions of livelihoods and economic processes and has been referred to as the heart of Africa, reflecting its societal importance and influence on the surrounding ecosystems. Many people have migrated to the lake shore over time, leading to an important transformation of societies around the lake who depend directly and indirectly on Lake Victoria for fish protein, economic livelihoods, transport, climate regulation, cultural values and other ecosystem services [5,11,[14], [15], [16]]. The lake supports the largest freshwater fishery in the world, producing 1 million tons of fish per year, including 230,000 tons of landed Nile perch that generate substantial foreign revenue [16,17], employing around 200,000 people, and supporting the livelihoods of nearly 45 million people [16,18]. In the surrounding basin, fish industries have been expanded and a limited number of new species have been introduced into the lake, which has changed ecosystem interaction of the flora and fauna in the lake [[19], [20], [21]]. This changing diversity of fish species and terrestrial anthropogenic inputs are driving environmental degradation of lake water [[22], [23], [24], [25], [26], [27], [28]]. Unrestricted fishing and overfishing caused by increasing population size and an international fish market [29] are changing lake ecosystems, leading to depletion of endemic species [19,20,30,31]. This in turn impacts tourism in the area which is of great economic importance locally and for international tourist companies (see Fig. 1). Tourism around Lake Victoria, especially parts of the industry framed as “eco-friendly”, relies on the conservation of the lake's natural and cultural heritage [14].

The deeper parts of Lake Victoria have been anoxic since 1990−1991 and almost half of the lake is affected by phosphorus accumulation, leading to saturation of oxygen levels. Chlorophyll concentrations and phosphorus deposition have increased 10-fold since the 1960 s [[32], [33], [34], [35], [36]]. Phosphorus is a key component of fertiliser that is intimately involved in terrestrial and marine biogeochemical cycles [37,38] and has been recognised as a key factor responsible for eutrophication in lake, estuarine and some other aqueous environments worldwide [39]. Atmospheric deposition also occurs, with soil particles, dust, and fires in the vicinity of the lake being the main sources of phosphorus in the atmosphere. Excessive inputs of phosphorus (mainly of anthropogenic origin) to water systems induce degradation of water quality through spurring the proliferation of algae, thus hindering various water uses [40]. Many factors, such as point and non-point source pollution, agriculture production, water hyacinths, industrialisation, the introduction of species and additional economic activities have increased nutrient loading to Lake Victoria. This is turn has led to eutrophication and increased turbidity, resulting in deterioration of lake water quality [36,[41], [42], [43], [44], [45], [46]].

The cumulative pressure from the surrounding basin environment is influencing the ability of Lake Victoria's ecosystems to cope with climate change [34,47]. Climate variability has been intensifying since the 1970 s, accompanied by changes in hydrology, aquatic ecosystems, and water balance of the lake. However, these changes have not been well documented for small and shallow aquatic systems, which are more vulnerable. Changes in temperature, rainfall and wind hydrodynamics around Lake Victoria need to be examined to provide better insights into how increasing climate variability and future climate change will affect water balance and lake water levels [48]. In summary, Lake Victoria's complex shallow water systems are influenced by social, economic, cultural, and ecological processes [49], which in turn affect social, economic, and environmental conditions for humans, animals, and plants in the region [50]. Therefore, within the wider governance of Lake Victoria's water quality, especially with intensifying climate variability, it is necessary to combine technical knowledge with data on social practices to ascertain the value of water of Lake Victoria. To support this, this study aimed to develop a hydrological model through finding a set of shallow water equations (SWEs) that can model water movement and pollution transport processes, a major socio-economic challenge for sustainable development. We tested shallow model concepts, including flow behaviour, wind hydrodynamics, different flow simulations and site-specific nutrient deposition, with the aim to identify suitable methods for investigating lake flow processes significant for water quality assessment. The aim was to create a method and model that can help assess the impacts of hydrodynamic processes, nutrient inputs, pollution transport processes, and eutrophication on the social and cultural water values of Lake Victoria.

There are previous important hydrodynamic models of Lake Victoria to which we contribute. This includes models based on thermodynamic and hydrodynamic characteristics from the Princeton Ocean Model (POM) 3D simulations [[51], [52], [53], [54]]. However, these models never considered the complex shoreline nor the real bathymetry of the lake but assumed an elliptic lake with surface wind stress to investigate the vertical lake temperature profiles. Nyamweya et al., 2016 [55] in turn used the Regional Oceanographic Model System (ROMS) to investigate how temperature and currents in Lake Victoria effect the diurnal, seasonal, and annual variations in stratification, vertical mixing, and inshore-offshore exchanges in the water column. USA's National Oceanic and Atmospheric Administration (NOAA) contributed a model for Lake Victoria with meteorological data as the first part of an integrated physical and ecological model with a focus on eutrophication [56]. Water level, pollution, eutrophication, and sediment flow analysis were partly also covered by Hecky and Kendall [57,58], but an elaborate bathymetry mapping combined with detailed hydrodynamics and analysis of climate effects on the dynamic processes that affect the lake's hydrology has previously not existed, which is what this paper contributes. In addition, this paper contributes a model that explicitly takes into account wind stress, which has not been modelled before despite being a key mechanism of shallow lakes affecting lake flow and wave motion and promote and shape pollution transport along the lake [59,60]. The need for our work is also supported by a growing regional and international interest in developing hydrological models to assess water quality in Lake Victoria, which has been expressed by the Lake Victoria Basin Commission (LVBC; see Ref. [61], who has published a series of reports since 2005 (e.g., LVBC Climate Change Adaptation Strategy and Action Plan, 2018–2023; LVBC Operational Plan 2015–2020 and Lake Victoria Environmental Management Project III.

Given the complex water system of Lake Victoria and the highly interconnected nature between the lake system, societal and industrial needs, and the impact of a changing climate, our study focused on specific water system components (highlighted in brown and green in Fig. 1) to reach a robust shallow lake water system model for assessing water quality. In the following section 2, we describe how we addressed our specific objectives, which were to: (i) illustrate and explore how a simple SWE solution for Lake Victoria behaves; (ii) connect this simple SWE-solution with a vertically integrated SWE numerical model system; and (iii) assess the model's predictions of total atmospheric source of phosphorus accumulation in the lake. Throughout, and building on previous publications [5,11], we used a 51 year empirical data series of precipitation, evaporation, in- and outflows of the lake, wind data from the Lake Victoria Basin Commission (LVBC) and phosphorous deposition measurements from 1996to2000 [33]. Results are given in section 3, followed by a Discussion (section 4) and Conclusions and recommendations (section 5). For an effective overview of our study, see Figure SFC1 and SMR1 in the beginning and end of the Appendix.

## 2 Materials and methods

### 2.1 Phosphorus deposition

**Fig. 2:**
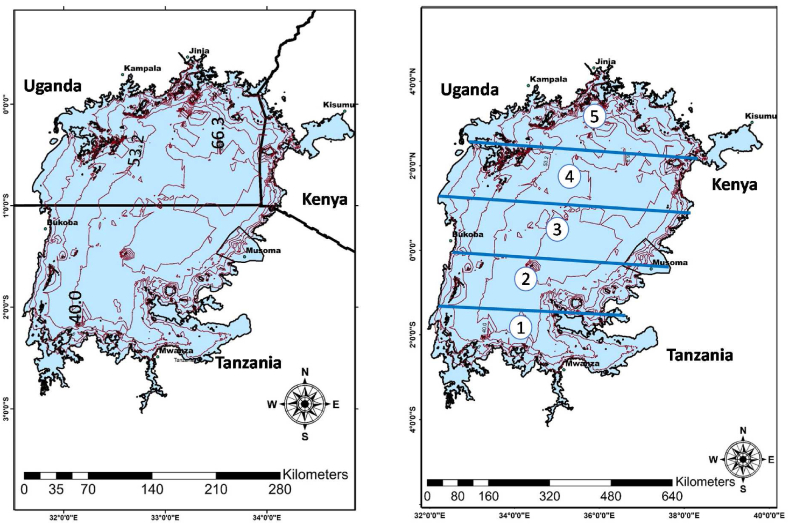
Maps showing (a) Lake Victoria model with lake depths contour lines, and (b) site-specific and section-wise phosphorus deposition in the lake.

For the site-specific phosphorus deposition model with lake depth (Fig. 2a) of Lake Victoria we include phosphorus entering the lake through precipitation, which changes across the wet and dry seasons [33]. Since the final estimate of lake-wide total atmospheric deposition will depend on the sampling site chosen, in this study the lake was divided into five horizontal phosphorus deposition sections to which empirical measurements were linked (Fig. 2b). A flowchart of the methods used for building the hydrological flow modelling and verifying it with empirical data from the LVBC [3,5] and Tamatamah et al. (2005) has been added in the supplementary section (**Flowchart SFC1**, **Supplementary Flowchart SFC1**). This includes the methods that were combined to improve the accuracy of empirical data of lake area-weighted estimates of phosphorus deposition rates that fed the model.

### 2.2 Shallow water wave equations

A one-dimensional (1D) depth-integrated shallow water system were then built where the bottom bathymetry B(x) was described in SWE by using the space variable x and the time variable t in the Cartesian coordinate system (x,t) [62,63]. The SWE describes a thin layer of fluid of constant density in hydrostatic balance, bounded from below by the bottom topography and from above by a free surface (Fig. 1, Supplementary Figure SF1). Bathymetry is one of the key parameters when modelling shallow lakes [5]. Lake Victoria is a shallow water body 250km in length (L), and 80m in depth (H), giving a lake shallowness of (H/L)=0.00032≤0.05 which is far below the standard shallowness parameter of lakes in the world (Equation 1 Supplementary Equation SE1).

Shallow water equations are formulated from the Navier-Stokes (N–S) equations, which are derived in turn from the principles of conservation of mass and momentum [64]. Two-dimensional (2D) vertically averaged SWEs have been applied to many water bodies where the water is assumed to be vertically mixed [[65], [66], [67], [68]]. In narrow and deep lakes, reservoirs, and estuaries, 2D laterally averaged models have sometimes been used as shallow water wave solutions [69]. In this study, the SWE is considered as a set of hyperbolic partial differential equations (PDE) and numerically solved by the finite volume method (FVM) and the finite element method (FEM), which describes the problem of fluid dynamics [62]. This is used to obtain the smooth solution for the sought-after surface hydrodynamics. For FVM, we used a higher-order resolution scheme for SWE on a rectangular mesh grid and established a raindrop diffusion model to initialise further lake surface flow assessment. For FEM simulations, a 2D vertically integrated SWE model was used where vertical velocity of lake water was assumed to be dependent on lake depth (h) and horizontal velocity u(x,t) was assumed to be roughly constant throughout the lake. The Comsol Multiphysics software, which we used, contains a FEM package for numerical solution of PDE that permits very accurate approximations of multiphysics field problems. Comsol uses both rectangular and triangular mesh grids and are very effective in using smoothing and geometry grid shapes to resolve necessary surface curves [70,71].

### 2.3 Conservation form for wave propagation SWEs

**Fig. 3:**
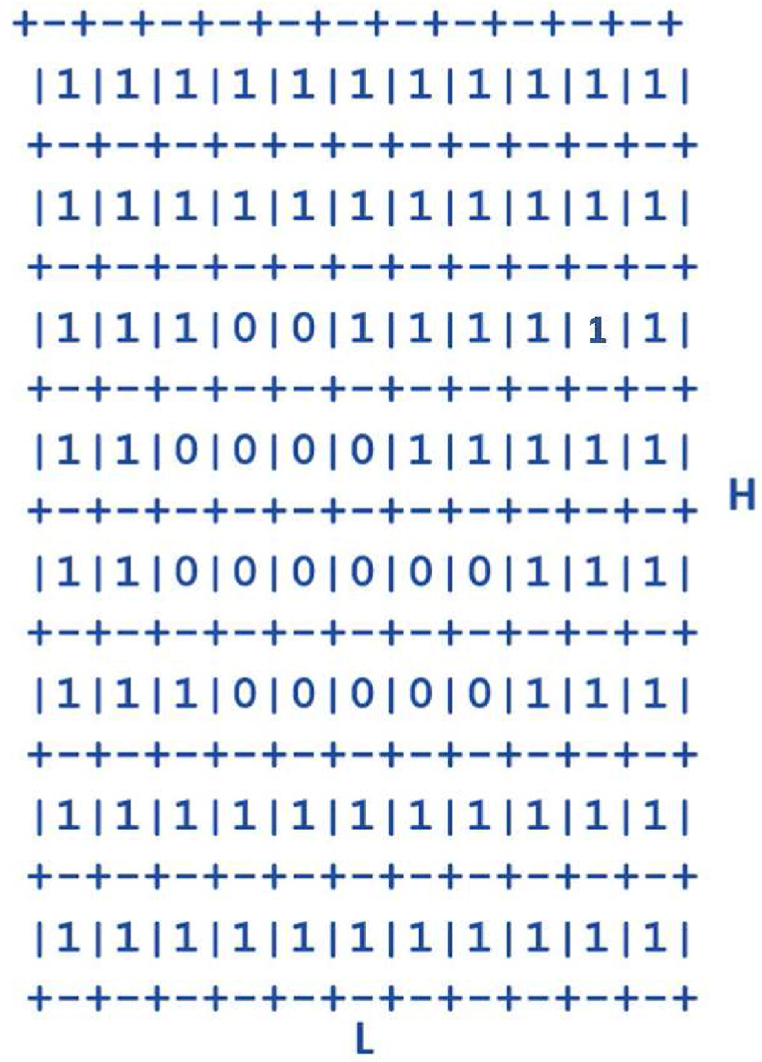
Geometric structure for modelling wave motion or advective transport of rain drops in a fluid region (this is also the geometric structure used for Fig. 4).

To also account for the irregular Lake Victoria shoreline geometry in our hydrological model, we started with the highly simplified geometry of a square (Fig. 3; see similar approaches: [[72], [73], [74]]. Given this simple geometric structure, and as explained in our previous study [5], an incompressible Newtonian equation was applied to 2D SWE where depth integration was achieved through disregarding dynamic effects in the vertical direction, (i.e., diffusion of momentum due to viscosity and turbulence, wind effects, the Coriolis term etc.). From this, we could compute the exact solution of raindrop diffusion in a fluid region by allowing the finite volume method (FVM) to consist of SWEs with two arbitrary waves (left and right states), where the concentration of the passive tracer (v) (the raindrop) moves with water velocity u, which tracks the tracer's motion in the simplified square geometric region (Fig. 3).

**Fig. 4:**
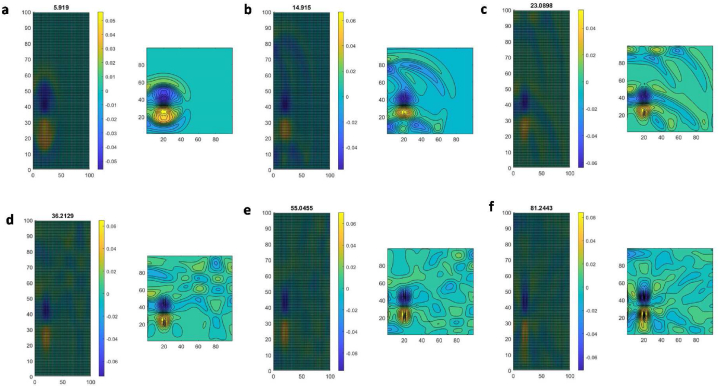
Default raindrop model obtained by applying a flux limiter. Water colour drop when diffused at (a) t=5.929s, (b) t=14.915s and (c) t=23.0898s and advective flow diffused with the whole bathymetry at (d) t=36.2129s, e) t=55.0455s and f) t=81.2443s. (For interpretation of the references to colour in this figure legend, the reader is referred to the Web version of this article.)

The default geometry for the raindrop model followed the structure of a box model matrix (with flow (1) and no-flow (0)), where flow refers to where water is present at the location and no-flow refers to where no water is present at the location, for instance where there is an island or a bay (Fig. 3). When data are discontinuous, the matrix goes to zero (0) and it becomes a first-order scheme. When the data are smooth, the matrix goes to one (1) and it becomes a second-order scheme. We set box model length (L) and width (H) to 100m (Fig. 3, Supplementary Method SM1).

### 2.4 Model set-up for SWEs under PDE

We have now arrived at the simple 2D vertically integrated SWE model developed for lake flow testing in Comsol (using FEM) for Lake Victoria (with and without islands). Given the equations of conservation of mass and conservation of momentum, the hydrological model takes the following form:

(1) $$u_{t}+uu_{x}+vu_{y}+g\left(h_{x}+b_{x}\right)=\mathrm{div}\left(\upsilon_{A}\nabla u\right)+fcx-C\left(h\right)V\;v_{t}+uv_{x}+vv_{y}+g\left(h_{y}+b_{y}\right)=\mathrm{div}\left(\upsilon_{A}\nabla v\right)+fcy-C\left(h\right)V\;h_{t}+{\left(hu\right)}_{x}+{\left(hv\right)}_{y}=\mathrm{div}\left(\upsilon_{h}\nabla h\right)$$

where

$$V=\sqrt{u^{2}+v^{2}},\nu_{A}=\mu \left(\sqrt{gh}+V\right)∙\Delta ,\upsilon_{h}=\upsilon_{A}+\Delta /\left(h+0.01\right)\text{,}$$

$$C\left(h\right)=ff/{\left(1+h/hhthin\right)}^{3},b\left(x,y\right)=-ampl/\sqrt{H^{2}-{\left(x-x_{0}\right)}^{2}-{\left(y-y_{0}\right)}^{2}}$$

Note that this is the non-conservative formulation for the primitive variables h, u and v. The conservative formulation in terms of the conserved quantities h (mass conservation), hu and hv (momentum conservation) is only needed if shocks (so called hydraulic jumps) appear.

**Fig. 5:**
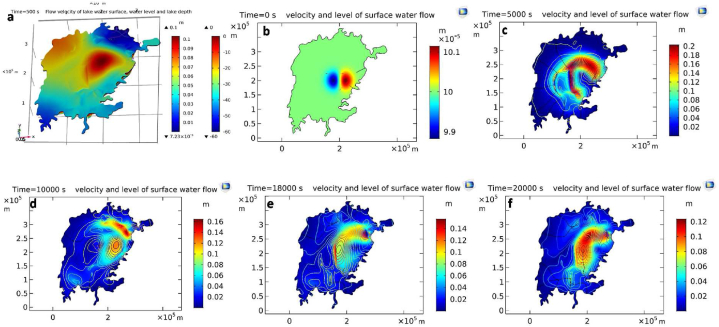
Flow model of Lake Victoria produced in Comsol. (a) Lake depth and lake level, where the shallow water equations (SWEs) adopted initial boundary conditions to visualise the raindrop diffusion in the lake; and lake flow reached at **(b)** t = 0s, **(c)** t = 5000s (1 h23 m), **(d)** t = 10000s (2 h46 m), **(e)** t = 18000s (5 h) and **(f)** t = 20000s (5 h33 m).

The difficulty in surface water modelling is to accurately resolve the effects of geometric boundaries. A model grid is a network of grid cells or points covering a specific area of a numerical model. To get an accurate hydrodynamic simulation, the grid representation of the boundary geometry should be as realistic as possible, following the lake shore and bathymetry of Lake Victoria. In lake bathymetry modelling in Comsol, a maximum depth of 60m was used, based on data obtained in preliminary model testing in Delft3D (Fig. 5). The coefficient form of PDEs in the model included initial flow velocity (very small), streamline diffusion, artificial viscosity, Coriolis forces, bottom friction etc., with boundary conditions (Supplementary Table ST 1). Given the above, the resultant lake free surface numerical model in the Comsol Multiphysics software was implemented using bathymetry and vertically integrated 2D shallow water equations, which were validated based on fifty year data series for lake water level and outflow as shown in our previous publication [5].

### 2.5 Wind hydrodynamics

Wind-driven flows are important to account for in shallow lakes [75]. The wind hydrodynamics assess model has been added in our validated vertically integrated SWE model. The wind speed increases with height in the atmospheric boundary layer, created by friction against the surface. The transmission of momentum between wind and water is generally formulated as shear stress (τ), depending on wind velocity with respect to water velocity, the density of air and a drag coefficient [76,77]. The standard wind measurement $W_{10}$ is made at 10m above the lake level [78]. The drag coefficient $C_{d}$ is indicative of the mean wind speed vertical gradient and increases with water surface roughness:

(2) $$\tau =\rho_{air}C_{d}\left(\left|W_{10}\right|\right)\left|\nu \right|\underline{\nu }$$

This model is only valid for small wind-induced waves, i.e., $W_{10}<12m/s$.

The wind stress can be expressed as:

(3) $$\tau =\left(F_{E},F_{N}\right)=\rho_{air}∙C_{d}∙\sqrt{v_{E}^{2}+v_{N}^{2}}∙\left(v_{E},v_{N}\right)$$

**Fig. 6:**
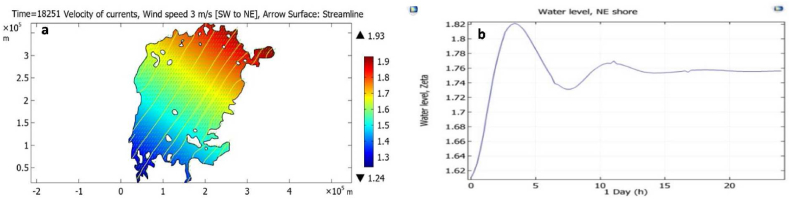
One-day constant wind speed simulation for Lake Victoria assuming a southwest wind, force 3m/s. (a) Water level at the end of the day and (b) water level variation at the northeast shore during the day.

Table 1 provides wind stress model parameters for Lake Victoria and Fig. 6 provides modelled output data results.

**Table 1:** Wind stress model parameters for Lake Victoria.

Parameter	Units	Value	Description
τ	$N/m^{2}$		*Surface shear stress*
$\rho_{air}$	$Kg/m^{3}$	1.225	*Air density*
$\rho_{water}$	$Kg/m^{3}$	1000	*Water Density*
$\underline{v\;}=\left(v_{E},v_{N}\right)$	m/s	$\left(W_{10}E-U,W_{10}N-V\right)$	*Lake surface velocity*(U,V) $W_{10}$*,*10m*above the mean water surface*
$C_{d}$	−	$\left(0.8+0.065∙W_{10}\right)∙10^{-3}$	*Friction coefficient*
$W_{10}$	m/s	$\sqrt{{\left(W_{10}E\right)}^{2}+{\left(W_{10}N\right)}^{2}}$	*Wind vector*$\left(W_{10}E,W_{10}N\right)$

### 2.6 Assessing whole lake phosphorus deposition

The whole lake phosphorus deposition in Lake Victoria was estimated by fitting a simple deposition equation to empirical data (for the equation used, see Supplementary Method SM 2). The empirical data used was atmospheric deposition samples collected from sites on the east, west, north, and southern shores of Lake Victoria by Tamatamah et al. (2005) and LVBC. Wet and dry atmospheric samples were collected using plastic buckets with 25.5cm top diameter and 30cm deep where “dry deposition samples were collected on a 2-week interval and wet deposition sampling on a storm event basis” for 6to12 months in 1999to2000 ([33]: 329) and coded as concentrations during 24 hours (μmol/(L∙day)). The rainfall data for each section of Lake Victoria (Fig. 2) were based on average rainfall in the five-year period 1996−2000. Using statistical analysis and calculations as provided in the supplementary section, the mean wet and dry deposition for each lake section was first calculated and used for calculating the entire lake-wide atmospheric total phosphorus (TP) deposition (Supplementary Method SM3-SM4). The final estimate of TP (in tonne per years) was computed by averaging phosphorus depositions recorded. Note that atmospheric deposition rates measured at Mwanza on the southern banks was excluded because of frequent contamination of samples with dust from unpaved city roads near the collection site [33]. The full method results have been added in the supplementary section (Supplementary Method Results SMR 1).

## 3 Results

### 3.1 Raindrop diffusion model in simplified geometric structure

A model for the simplified geometric box-structure of the lake was first developed using Matlab (for the code, see Supplementary Method SM1). To produce a high-resolution model without spurious oscillations often associated with right- and left-moving diffusion, a limitation on water flux was used (both second- and first-order)(Fig. 4a–f). The maximum timescale was set to 82s to illustrate the flow behaviour in the geometrically artificial square lake. The flow was started from the initial time step and spread out within 23s. When the flow expanded after 36s, an adverse pressure gradient was encountered as the flow expander caused an extended region of dispersive flow. The flow dispersal continued until 82s, whilst the contour function was working properly. There was little effect on the water level from the diffusion limiter. Therefore, flow diffusion in a cell-model worked successfully under a FVM high-resolution scheme.

### 3.2 Lake flow model using realistic lakeshore boundaries and bathymetry

An accurate lake hydrodynamic model requires a mesh grid that is as realistic as possible. In modelling the real and much more complex shoreline of Lake Victoria, we used the interpolation function in Comsol to interconnect mesh grid points to build lakeshore boundaries and lake bathymetry (Fig. 5). To run the model, the initial boundary condition has used on eq. (1) with flow of raindrop diffusion starting from the beginning (at t=0s) and in an eastern point in the middle of the lake (Fig. 5a) and simulating its dispersal with increasing time (Fig. 5b–f).

The lake surface water flow changed the lake water level unless the lakeshore was very steep. Use of moving grid with a normal speed of u.n made insignificant lake surface water flow and shoreline movements. In the Lake Victoria model, no-flow was used as the outer boundary and artificial diffusion was added in the depth equation (assuming that depth (h) was small), which in turn exhibited significant diffusion. When h was started at non-zero everywhere and the actual shoreline was modelled as an isoline (contour line), h never vanished. To avoid a normal wave at the shore, we turned on a friction coefficient where h was small. The flow velocity in the thin layer can disperse both upstream and downstream and we observed how smooth solutions appeared. Fig. 5 shows how the velocity of surface water flow in Lake Victoria increased with respect to time and the lake water level changed with lake depth. After a certain period, the surface water flow became placid and came back to the initial stage with a dissipative mode (Fig. 5b–f). Proper river flow boundary conditions must be imposed for a realistic lake hydrodynamic model.

### 3.3 Wind model simulation

Wind-driven flows (Eqs. (2), (3)) of the lake based on wind velocities can be much larger than those created by localised in- and outflows from in- and out-going rivers. In temporally long steady wind forcing, the water level gradient approached the $W_{10}$ wind direction, and the water level difference across the lake was proportional to $W_{10}^{2}$, as seen from the expressions in Table 1.

Fig. 6a shows the effect of a3m/s southwest wind, “turned on” in the model and kept constant thereafter, while Fig. 6b shows how this wind created a *seiche*, i.e., damped sloshing of the whole lake with a period of about 6 hours. This is consistent with the estimate of 3.5hours for the time it takes for a gravity wave to cross the lake. Artificial viscosity and bottom friction are the dissipative processes responsible for damped surface motion. Thus, a steady wind makes the velocity gradient balance the associated wind stress, with little effect on flow velocities driven by transient inflows and outflows. It follows that temporally and spatially resolved wind field data are required to determine the true influence of wind on flow patterns.

### 3.4 Modelling spatial distribution of total phosphorus deposition

**Fig. 7:**
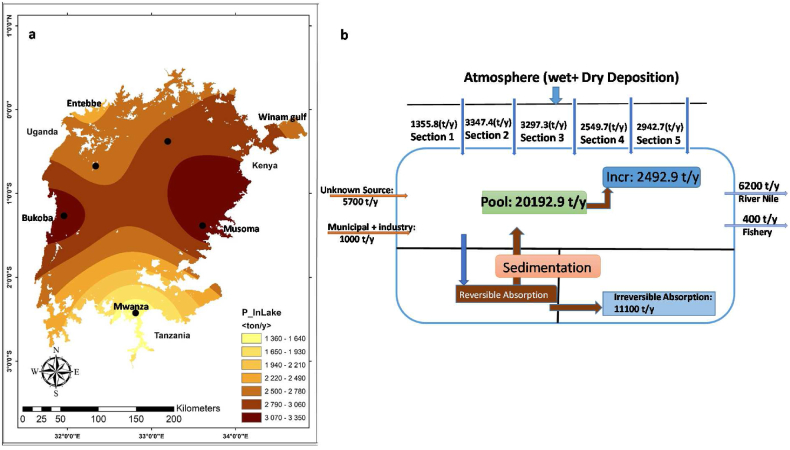
(a) Modelled spatial distribution of atmospheric phosphorus (P) deposition in Lake Victoria and (b) results of total mass-balance calculations.

Combining the models developed in 3.1,3.2 and 3.3, thus considering a realistic shoreline, bathymetry, and wind-driven flows, the spatial distribution of atmospheric phosphorus deposition across wet and dry seasons, can now be modelled (Fig. 7a). The input to this model was the measured daily mean wet and dry atmospheric total phosphorus (TP) deposition at the different sampling sites as presented in the Appendix (Supplementary Method SM2- SM4; Supplementary Table ST 2**)**. This accounts for differences in mean annual amounts from dry and wet season days between the five different sections of the lake. As already explained above, we divided Lake Victoria horizontally into five sections that were aligned to the atmospheric deposition measurement by Tamatamah et al. (2005) and LVBC along the shore of the lake. Each section of Lake Victoria thus contained one measurement site (Fig. 7a), which combined were used to provide data on phosphorous deposition across the whole lake (Fig. 7b).

The results from measured data show that annual TP deposition from the atmosphere is about 13,500 tons with most of the TP deposition (69%) coming from storms in the dry period. Based on the annual deposition values, there was a yearly surplus of phosphorus, increasing the concentration in the lake by 2492.9 tons annually. When running the hydrological model the total phosphorus concentration ranged between 1350to3300ton/year (Fig. 7a) and was seen to increase in higher concentrations at the Bukoba and Musoma sites, lying midway across the lake on the west and east shore, respectively. Lower concentrations were modelled to become concentrated south of Bukoba and Musoma with the lowest concentration in the far south in the Mwanza Gulf (Tanzania). In the north, both Entebbe (Uganda) and the deep Winam Gulf (Kenya) recorded lower values. The model results show relatively good agreement with previous phosphorus estimations reported by Garcia-Navarro and colleagues in 2019 [78] who used similar sampling sites.

## 4 Discussion

For the complex lake water system in Lake Victoria, we used SWEs to solve realistic situations involving realistic shoreline boundaries and assessing wind flows based on empirical data. A higher-order resolution approach was adopted for assessing raindrop diffusion and vertically integrated shallow water flows were applied for assessing dynamic flows in Lake Victoria. To improve the model, the hydrostatic balance of shallow-water flows was calculated when assessing wind hydrodynamics. Both flow- and wind-driven analysis, including initial values for the dependent variables, can serve as an initial condition for a transient simulation. As demonstrated in this study, the difference between predicted high-resolution SWE and vertically integrated SWE was significant for water flow-induced changes, where diffusion and dispersion play a major role. SWEs have been widely used for open channel flow, tsunami, and flood modelling [79], and the present study demonstrated that they are also suitable for shallow lake simulations and corresponding lake hydrodynamics. One drawback of the approach was the high computational effort required to obtain accurate numerical simulations.

There is increasing evidence that the water level and ecosystem in shallow lakes are affected by nutrient loading such as phosphorus [80,81] and that wind speed influences nutrient inflows into shallow lake water systems [82]. Higher wind speed causes wind waves, which promote nutrient release to the water column and enhance release of bottom nutrients from sediment. Lake floor sediment is furthermore an important habitat in aquatic ecosystems [83] and Lake Victoria has experienced increasing phosphorus enrichment within both its sediments and water column [32,78,84,85]. Deposition of essential plant nutrients, such as nitrogen (NO_3_–N) and phosphorus (PO_4_–P), has enhanced the nutrient load in the shallow lake water system of Lake Victoria [86]. Tamatamah et al. (2005) estimated that atmospheric deposition represents 55% of the total phosphorus input to Lake Victoria (Fig. 7b). Therefore, qualitative research can rely on measuring total phosphorus (TP) accumulation (wet + dry) in the lake and use our model to combine these measurements with mass balance calculations for different horizontal lake sections. Our model indicates that of the 13,500 tons of measured annual atmospheric phosphorus deposition, water currents concentrate phosphorus in the western and eastern central lake regions, while concentrations of phosphorous were lower in the north and south of the lake. Our modelled phosphorus accumulation results were similar with the total phosphorus mass balance calculations despite the different methods that were used, showing clear similarities in the detection of higher concentrations in specific lake regions.

While we acknowledge that the assessment of a single nutrient is not sufficient to assess water quality and lake eutrophication, our model can be combined with data from previous studies. Gukuma-Njuru [78], for instance, measured total phosphorus and other soluble nutrient concentrations along the lakeshore and inlets of Lake Victoria and Njagi et al. [87] analysed the combined role of nutrients and land use influences on lake eutrophication. Future work could combine our model with studies like these and compare all nutrient concentrations in the lake water system separately and assess their individual influence on water quality. Using bio-geochemical analysis, this could further generate a model of overall lake nutrient concentration to improve lake water management. To strategically improve future studies, lake modelling and measurement in Lake Victoria should focus on shallow lakeshore areas that are strongly affected by human activities that cause and modify lake water nutrient influxes and are key sites where critical ecosystem services and drinking water supply can be protected.

## 5 Conclusions and recommendations

In this study, SWEs were chosen as a primary test for developing more realistic hydrodynamic models of Lake Victoria. The study drew upon numerical modelling (FVM and FEM) and used a default raindrop diffusion model to avoid “shaking motions”. To get a proper surface flow model (i.e., flow velocity and water level), a vertically integrated SWE model was used. The surface flow model developed showed that the prevailing wind moves from southeast to northwest in Lake Victoria. Yet, the analysis of hourly data for one day revealed that the lake water level increased by almost 200mm within 6hours of wind and reached steady state again 10hours later. When it comes to the deposition of phosphorus to the lake, the total modelled estimate was 13,500tons, which can be considered a reasonable value based on the literature. There are however many contributors to eutrophication of Lake Victoria, of which phosphorus deposition from the atmosphere, which was modelled here, is only one. Soil particles, dust, and fires in the vicinity of the lake are the major sources of phosphorus in the atmosphere but such fluxes, which are likely to increase if biomass burning and deforestation continue, are hard to take into account and require further investigations.

In our previous investigations, we have developed a fully integrated 2D SWE model in Comsol. We have used extensive data to smooth the lake topography, which has improved the reliability and precision of our hydrological model. In the future, we plan to incorporate total lake nutrients and pollution transport in the integrated lake model to better evaluate the water quality of the lake. Such a fully developed lake hydrodynamic model can be validated against empirical measurements of mean lake water flow-level and become an even more precise tool for the natural resource management of lake *Nalubaale* as the “abode of the spirits”.

## Data availability

The Comsol Multiphysics (Comsol 2015; Comsol 2019) software has many model libraries to focus on different areas, but no special model libraries for surface water body analysis. We collaborated with Makerere University, Uganda. Matlab was used for bathymetry data processing. Lake bathymetry data and lake flow data have been collected from LVBC and NaFIRRI (See AUTHOR et al., 2019). Model parameters and mass-balance calculations are presented in an appendix to this paper.

## Additional information

No additional information is available for this paper.

## CRediT authorship contribution statement

**Seema Paul:** Writing – review & editing, Writing – original draft, Visualization, Validation, Methodology, Funding acquisition, Formal analysis, Data curation, Conceptualization. **Benedict T.I. Reinardy:** Writing – review & editing, Writing – original draft, Investigation, Conceptualization. **David Ddumba Walakira:** Data curation. **Prosun Bhattacharya:** Writing – review & editing. **Henrik Ernstson:** Writing – review & editing, Supervision, Investigation. **Zahra Kalantari:** Writing – review & editing, Supervision.

## Declaration of competing interest

The authors declare that they have no known competing financial interests or personal relationships that could have appeared to influence the work reported in this paper.
