# Supplementary material for: A shallow water numerical method for assessing impacts of hydrodynamics and nutrient transport processes on water quality values of Lake Victoria
Source: Heliyon. 2024 Feb 3;10(3):e25125. doi: 10.1016/j.heliyon.2024.e25125 (PMC10862508; doi:10.1016/j.heliyon.2024.e25125)
Supplement: Multimedia component 1 [file mmc1.docx]

**Appendix A. Supplementary data**

**A shallow water numerical method for assessing impacts of hydrodynamics and nutrient transport processes on water quality values of Lake Victoria**

Seema Paul^1, 2, *^, Benedict T.I. Reinardy^1^, David Ddumba Walakira^4^, Prosun Bhattacharya^1^, Henrik Ernstson^3^, Zahra Kalantari^1^

*^1^Division of Water and Environmental Engineering, Department of Sustainable Development, Environmental Science and Engineering, KTH Royal Institute of Technology, Teknikringen 10B, 114 28 Stockholm, Sweden*

*^2^Numerical Analysis, CSC Skolan, KTH Royal Institute of Technology, Stockholm, Sweden*

*^3^Division of Strategic Sustainability Studies, SEED, KTH Royal Institute of Technology, Stockholm*, Sweden

*^4^Mathematics Department, Makerere University, 7062 Kampala, Uganda*

* Correspondence author:

*E-mail address:* [seemap@kth.se](mailto:seemap@kth.se) (S. Paul)

**Table of Content**

**Supplementary Flowchart : SFC1** Total methodology flowchart of the research paper.

**Supplementary Figure: SF1** One-dimensional shallow water wave equation (SWE) model.

**Supplementary Equation: SE1** One-dimensional shallow water wave equations (SWE).

**Supplementary Method: SM1** Shallow water flows modelling by a 2D high-resolution scheme.

**Supplementary Table: ST1** Vertically integrated Shallow water Equation 2D model parameters.

**Supplementary Method: SM2** Phosphorus deposition in Lake Victoria (dry and wet season).

**Supplementary Method: SM3** Phosphorus deposition in Lake Victoria (dry season).

**Supplementary Method: SM4** Phosphorus deposition in Lake Victoria (wet season).

**Supplementary Table: ST2** Data and results of dry, wet and dry+wet season phosphorus depositions in the five sections of Lake Victoria used for total phosphorus (TP) deposition calculation.

**Supplementary Method Results: SMR 1** Overall method results of the research paper.

**Flowchart SFC1**

**Supplementary Flowchart: SFC1** Total methodology flowchart

**Flowchart SFC1.** The total methodology used to build the hydrological flow model of Lake Victoria—shallow water, vertically integrated, and wind-driven flow—is given in the above flowchart with equations. The vertically integrated 2D shallow water model was verified with measured outflow and water levels for the last 51 years by the Lake Victoria Basin Commission (LVBC; see AUTHOR et al. 2019). The phosphorus deposition model was verified by data from Tamatamah et al. (2005) who measured phosphorous deposition in the lake from 1996 to 2000.

**Supplementary Figure SF1 and Supplementary Equation SE1.**

**Supplementary Figure SF1.** One-dimensional shallow wave equations (SWE), where $\eta$ is wave height above the surface, $h$ is total water depth and B is bathymetry. (Left) Actual bottom geometry of Lake Victoria in the SWE model and (right) flat bottom topography in numerical simulations.

The mass conservation continuity equation gives (**Supplementary Equation SE1**):

| $\frac{\partial u}{\partial x}+\frac{\partial v}{\partial y}+\frac{\partial w}{\partial z}=0$ | (1) |
| --- | --- |

For Lake Victoria, the vertical momentum exchange is negligible and the vertical velocity component $w$ is smaller than the horizontal components $u$ and $v$: $w\ll u$ and $w\ll v$

$$\mathcal{O}\left( \frac{U}{L} \right)\mathcal{O}\left( \frac{U}{L} \right)\mathcal{O}\left( \frac{W}{H} \right)=0$$

$$\Rightarrow W\sim U\frac{H}{L} with \frac{H}{L} <<1$$

Therefore, Lake Victoria is a shallow water body where the horizontal scale of motion is much larger than the vertical scale.

**Supplementary Method SM1: Shallow water flows modelling by a 2D high-resolution scheme**

**% shallow water eqns Roe scheme 2D**

**%=====================================================**

**% ... the non-rect geometry does not work ...**

**% uses:**

**% roestep2D**

**% setbc2D**

**% roeflux2D**

**% SWEfluxX**

**% SWESRC**

**% limiter**

**%=====================================================**

**% ht + (m )x + (n )y = 0**

**% mt + (m^2/h+1/2gh^2)x + (mn/h )y = -ghbx**

**% nt + (mn/h )x + (n^2/h + 1/2gh^2)y = -ghby**

**% h m n**

**% JX = ( 0 1 0 ) h**

**% (-u^2+gh 2u 0 ) m**

**% (-uv v u ) n**

**% eigenvalues c = sqrt(gh)**

**% u-c u+c u**

**% vectors**

**% ( 1 1 0 )**

**% R = ( u-c u+c 0 )**

**% ( v v 1 )**

**% L = R^(-1):**

**% ( u+c -1 0 )**

**% L = (-u+c 1 0 )*1/(2c)**

**% (-2vc 0 2c )**

**% Roe average**

**% hbar = 1/2(hi + hi+1); sqrt(hi) = ki**

**% uhat = (ki ui + ki+1 ui+1)/(ki + ki+1)**

**% vhat = (ki vi + ki+1 vi+1)/(ki + ki+1)**

**% chat = sqrt(g*hbar);**

**% Atilde = JX(hbar,uhat,vhat)**

**% |Atilde| = R*diag(abs(uhat-chat) abs(uhat+chat) abs(uhat)]*L**

**%**

**%==== different ordering for Jy**

**% h n m**

**% J2Y = ( 0 1 0 ) h**

**% (-v^2+gh 2v 0 ) n**

**% (-uv u v ) m**

**%==== makes it possible to use the 1-D x-direction program:**

**% J2Y(h,u,v) = JX(h,v,u)**

**% A2tilde = JX(hbar,vhat,uhat)**

The total depth $(h)$ and the velocity vector $(u,v)$ have been considered to account for the artificial water flow patterns in FVM, $g=9.81m/{s^{2}}, h_{in}=1m, u_{in}=0m/s, v_{in}=0m/s, h_{out}=1m, u_{out}=0m/s, v_{out}=0m/s$.

**Table ST1.** Parameters used in Comsol Multiphysics for model simulations

| **Parameter** | **Units** | **Value in Comsol** | **Expression** |
| --- | --- | --- | --- |
| **Differential equations** | | | |
| $x, y$  $u, v$  $\Delta$  $h$  $g$  $\mu$  $C(h)$  $fcx, fcy$  $ff$  $hhthin$ | $m$ | $(6e5, 6e5)$ | *Velocity coordinates (east, north)* |
|  | m/s | $0.0001, 0$ | *Mean velocity of water* $(x, y)$ |
|  | m | $0.75$ | *Local element mesh size* |
|  | m | $2$ | *Depth of the water from the bottom to top of the surface water* |
|  | $m/s^{2}$ | $10$ | *Gravitational acceleration* |
|  | - | $0.4$ | *Artificial viscosity/diffusion coefficient* |
|  | $m^{-1}$ | $0.0001$ | *Wall friction coefficient* |
|  | $s^{-1}$ | - | *Coriolis parameter* |
|  | $m^{-1}$ | $100$ | *Friction coefficient for this water* |
|  | m | $0.01$ | *Thickness of water film of lakeshore* |
| **Boundaries (outer boundary)** | | | |
| $Vn_{x}+Vn_{y}$  $b(x,y)$  $ampl$  $H$  $x_{0}$  $y_{0}$ | $m/s$ | $0$ | *Wall normal velocity* |
|  | m | $60$ | *Bathymetry* |
|  | $m$ | $0.01$ | $O(1) m deep$ |
|  | $m$ | $125$ | *Maximum depth* |
|  | $m$ | $20$ | $x$ *at a maximum depth* |
|  | $m$ | $5$ | $y$ *at a maximum depth* |
| **Initial data** | | | |
| $h_{0}$  $V$ | $m$ | $0.2$ | *Initial water depth* |
|  | $m/s$ | $0$ | $Initial values for u \& v$ |

**Method SM2.** Phosphorus deposition in Lake Victoria (dry and wet season).

| ${TP}_{depo}=m*{PD}_{mean}*D*a$ | (2) |
| --- | --- |

where ${TP}_{depo} is total phosphorus deposition \left[ {tons}/{year} \right]$; $31$ is the molecular weight $(m)$ of $phosphorus$; ${PD}_{mean}$ is a mean deposition for wet and dry season and counted by Tamatamah et al 2005. Two types of procedure were used to estimate P deposition rates measurement to Lake Victoria, these are $wet=\left[ {\mu mol}/{(m^{2} day)} \right] and dry=\left[ {\mu mol}/{{(m}^{2} day)} \right]$; $a$ is total lake surface area; and $D$ is number of days in the dry-season and number of days in the rainy season.

**Method SM3.** Estimated dry phosphorus deposition to Lake Victoria. Illustrated by the first lake section. The same calculation is used for all five sections with results in **Table ST2**.

$${TP}_{depo}=m*{PD}_{mean}*D*a=31*24\cdot4*200*68.80*{10}^{8}{\mu g}/y=8.701*{10}^{14}{\mu g}/y=\frac{8.701*{10}^{14}}{{10}^{6}}g/y=870.18*{10}^{6}g/y=870.18*{10}^{3}{kg}/y=870.2{tons}/{years}$$

**Method SM4.** Estimated wet phosphorus deposition to Lake Victoria. Illustrated by the first lake section. The same calculation is used for all five sections with results in **Table ST2**.

$${TP}_{depo}=m*{PD}_{mean}*D*a=31*13\cdot8*165*68.80*{10}^{8}{\mu g}/y=4.85638*{10}^{14}{\mu g}/y=\frac{4.85638*{10}^{14}}{{10}^{6}}g/y=485.6*{10}^{6}g/y=485.6*{10}^{3}{kg}/y=485.6{tons}/{years}$$

**Table ST2.** Data and results of dry, wet and dry+wet season phosphorus depositions in the five sections of Lake Victoria used for total phosphorus (TP) deposition calculation.

| **Lake section** | **1** | **2** | **3** | **4** | **5** |
| --- | --- | --- | --- | --- | --- |
| Surface area, a | 6880 ${km}^{2}$  = 68.8*${10}^{8}m^{2}$ | 17,200 ${km}^{2}$  = 172*${10}^{8}m^{2}$ | 18,576 ${km}^{2}$  = 185.8*${10}^{8}m^{2}$ | 13,074 ${km}^{2}$  = 130.7*${10}^{8}m^{2}$ | 13,070 ${km}^{2}$  = 130.7*${10}^{8}m^{2}$ |
| ***Dry season*** |  |  |  |  |  |
| Mean deposition, ${PD}_{mean}$ | 24.4 | 20.4 | 17.4 | 20.4 | 21.6 |
| Dry season days/year, D | 200 | 205 | 240 | 190 | 250 |
| Phosphorus deposition, ${TP}_{depo}\left[ \frac{tons}{years} \right]$ | 870.2 | 2229.8 | 2404.7 | 1570.9 | 2187.9 |
| ***Wet season*** |  |  |  |  |  |
| Mean deposition, ${PD}_{mean}$ | 13.8 | 13.1 | 12.4 | 13.8 | 16.2 |
| Wet season days/year, D | 165 | 160 | 125 | 175 | 115 |
| Phosphorus deposition, ${TP}_{depo}\left[ \frac{tons}{years} \right]$ | 485.6 | 1117.6 | 892.6 | 978.8 | 754.8 |
| ***Wet + dry season*** |  |  |  |  |  |
| Mean deposition, ${PD}_{mean}$ | 24.4+13.8  = 38.2 | 20.4+13.1  =33.5 | 17.4+12.4  =29.8 | 20.4+13.8  =34.2 | 21.6+16.2  =37.8 |
| Day/year, D | 200+165=365 | 205+160=365 | 240+125=365 | 190+175=365 | 250+115=365 |
| Phosphorus deposition, ${TP}_{depo}\left[ \frac{tons}{years} \right]$ | 870.2+485.6  =1355.8 | 2229.8+1117.6  =3347.4 | 2404.7+892.6  = 3297.3 | 1570.9+978.8  = 2549.7 | 2187.9+754.8  = 2942.7 |

**Supplementary Method Results: SMR 1**
